# Bioinformatic analysis suggests that the Cypovirus 1 major core protein cistron harbours an overlapping gene

**DOI:** 10.1186/1743-422X-5-62

**Published:** 2008-05-20

**Authors:** Andrew E Firth, John F Atkins

**Affiliations:** 1 BioSciences Institute, University College Cork, Cork, Ireland

## Abstract

Members of the genus *Cypovirus *(family *Reoviridae*) are common pathogens of insects. These viruses have linear dsRNA genomes divided into 10–11 segments, which have generally been assumed to be monocistronic. Here, bioinformatic evidence is presented for a short overlapping coding sequence (CDS) in the cypovirus genome segment encoding the major core capsid protein VP1, overlapping the 5'-terminal region of the VP1 ORF in the +1 reading frame. In Cypovirus type 1 (CPV-1), a 62-codon AUG-initiated open reading frame (hereafter ORFX) is present in all four available segment 1 sequences. The pattern of base variations across the sequence alignment indicates that ORFX is subject to functional constraints at the amino acid level (even when the constraints due to coding in the overlapping VP1 reading frame are taken into account; MLOGD software). In fact the translated ORFX shows greater amino acid conservation than the overlapping region of VP1. The genomic location of ORFX is consistent with translation via leaky scanning. A 62–64 codon AUG-initiated ORF is present in a corresponding location and reading frame in other available cypovirus sequences (2 CPV-14, 1 CPV-15) and an 87-codon ORFX homologue may also be present in *Aedes pseudoscutellaris reovirus*. The ORFX amino acid sequences are hydrophilic and basic, with between 12 and 16 Arg/Lys residues in each though, at 7.5–10.2 kDa, the putative ORFX product is too small to appear on typical published protein gels.

## Findings

The genus *Cypovirus *(cytoplasmic polyhedrosis viruses; CPVs) is one of ≥ 12 genera within the *Reoviridae*, a family of segmented dsRNA viruses. While other members of the family infect mammals (e.g. *Bluetongue virus*), including humans (e.g. rotaviruses, coltiviruses, mammalian orthoreoviruses and seadornaviruses), CPVs infect insects. CPV species have been divided into 16 or more types (CPV-1, CPV-2, etc) based on electrophoretic migration of the genome segments [1]. Of the 352 *Reoviridae *RefSeqs in GenBank (10 Apr 2008; 33 species × 9–12 segments per species), only ~5% are multicistronic. Among these are a few examples of fully overlapping genes apparently translated via leaky scanning, for example in mammalian *Orthoreovirus *segment S1 [2], *Phytoreovirus *segment S12 or S9 [3], and (currently not experimentally verified) *Orbivirus *segment 9 [4]. Such overlapping CDSs can be difficult to detect using conventional gene-finding software [5], especially when short. The software package MLOGD, however, was designed specifically for identifying such CDSs, and includes explicit models for sequence evolution in double-coding regions as well as models for single-coding and non-coding regions [5,6]. Using MLOGD, we recently identified – and subsequently experimentally verified – a new short CDS in the *Potyviridae *family that overlaps the P3 cistron but is translated in the +2 reading frame [7]. When we applied MLOGD to the cypoviruses we also found evidence for a short overlapping CDS. Here we describe the bioinformatic analysis.

The putative new CDS (hereafter ORFX) was first identified in an alignment of the RefSeq [GenBank: NC_003016] with its CPV-1 genome neighbours. Subsequently, all homologous CPV sequences in GenBank were located by applying tblastn [8] to the NC_003016 VP1 amino acid sequence, resulting in the sequences [GenBank: AF389462] – *Lymantria dispar cypovirus 1 *segment 1 (CPV-1), [GenBank: AF323781] – *Bombyx mori cypovirus 1 *segment 1 (CPV-1), [GenBank: AY163247] – *Dendrolimus punctatus cypovirus 1 *segment 1 (CPV-1), [GenBank: AY388398] – *Bombyx mori cypovirus 1 *segment 1 (CPV-1), [GenBank: AF389453] – *Lymantria dispar cypovirus 14 *segment 2 (CPV-14), [GenBank: DQ388474] – *Heliothis armigera cypovirus 14 *segment 2 (CPV-14), [GenBank: AF291684] – *Trichoplusia ni cypovirus 15 *segment 2 (CPV-15), and [GenBank: DQ087278] – *Aedes pseudoscutellaris reovirus *segment 3 (APRV). APRV has only 9 segments and is not classified as a cypovirus [9], but was nonetheless included in the analysis. Note that the GenBank RefSeqs NC_003016, NC_003007 and NC_002558 were derived, respectively, from AF389462, AF389453 and AF291684.

**Figure 1:**
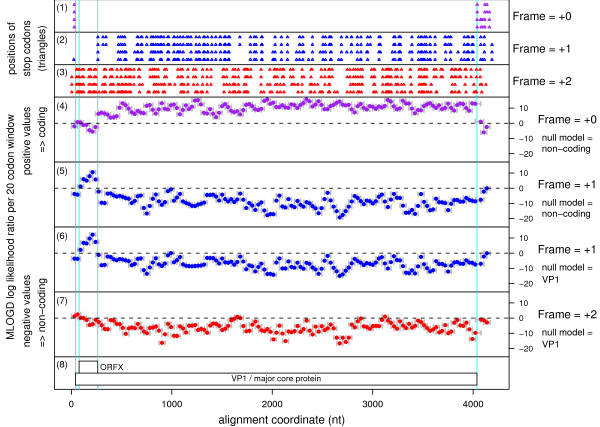
**MLOGD statistics for the alignment of four CPV-1 segment 1 sequences**. The four sequences were aligned with code2aln [15]; the alignment is gapless within the VP1 ORF. **(1)–(3) **The positions of stop codons in each of the four sequences in each of the three forward reading frames (frame defined by alignment to the reference sequence [GenBank: AF389462]). Note the conserved absence of stop codons in the +0 frame within the VP1 ORF and in the +1 frame in the ORFX region. **(4)–(7) **MLOGD sliding-window plots. Window size = 20 codons. Step size = 10 codons. Each window is represented by a small circle (showing the likelihood ratio score for that window), and grey bars showing the width (ends) of the window. See [6] for further details of the MLOGD software. In **(4)–(5) **the null model, in each window, is that the sequence is non-coding, while the alternative model is that the sequence is coding in the window frame. Positive scores favour the alternative model. There is a strong coding signature in the +0 frame (4) throughout the VP1 ORF, except where it overlaps ORFX. In this region there is a strong coding signature in the +1 frame (5) indicating that ORFX is subject to stronger functional constraints than the overlapping section of VP1. In **(6)–(7) **the null model, in each window, is that only the VP1 frame is coding, while the alternative model is that both the VP1 frame and the window frame are coding. Only the +1 (6) and +2 (7) frames are shown because the +0 frame is the VP1 frame which is included in the null model. Scores are generally negative with occasional random scatter into low positive scores, except for the ORFX region which has consecutive high-positively scoring windows (6). **(8) **Map of the reference sequence [GenBank: AF389462].

In the four CPV-1 sequences, ORFX has 62 codons (nt coords AF389462:77..262; 7.5 kDa) and overlaps the 5'-terminal region of the VP1 ORF (nt coords AF389462:40..4038) in the +1 reading frame (Figure 1). Here the VP1 ORF starts at AUG2 (context acgAUGc) while ORFX starts at AUG3 (context auaAUGa).

Interestingly, AUG1 (context [g/u]guAUGu; nt coords AF389462:11..13) is also in the ORFX frame and, in AY163247 and AF323781, could allow a 22-aa N-terminal extension of ORFX; however, in AF389462, there is an in-frame termination codon four codons 3' of AUG1 and, furthermore, the MLOGD results (Figure 1; see below) do not support the N-terminal extension.

In order to measure the coding potential of ORFX in CPV-1, we used MLOGD [5]. Applied to an alignment of the four CPV-1 sequences, MLOGD detected a strong coding signature for ORFX, with three non-overlapping – and hence completely independent – positively scoring windows in the ORFX region (Figure 1). The number of independent base variations across the alignment within the ORFX region is N_var _~ 33, and the total MLOGD score is log(LR) ~ 23.4 (see [6] for details). Extensive tests with known single-coding and double-coding virus sequence alignments indicate that 'N_var _≥ 20' and 'log(LR) ≥ $\frac{1}{6}$ × N_var_' signals robust detection (<1% false positive rate) of an overlapping same-strand CDS [6] (and unpublished data). Moreover, the MLOGD results showed that the ORFX amino acid sequence is considerably more conserved at the amino acid level than the overlapping region of VP1 (Figure 1). Indeed, in pairwise comparisons between AF389462 and each of the other three CPV-1 sequences, there was 92–100% amino acid identity in ORFX, but only 74–77% amino acid identity in the overlapping region of VP1.

In the two CPV-14 sequences, ORFX has 64 codons (nt coords DQ388474:70..261; 7.7 kDa) and overlaps the 5'-terminal region of the VP1 ORF (nt coords DQ388474:39..3947) in the +1 reading frame. In DQ388474, the VP1 ORF starts at AUG2 (context gauAUGu) while ORFX starts at adjacent AUG [34] (contexts aagAUGAUGa). AUG1 (context uagAUGa) at nt coords DQ388474:20..22 is in the *-*1 frame relative to the VP1 ORF and heads a 15-codon ORF terminating at a UAA codon which is separated from the ORFX AUG codon by a 2-nt spacer. In AF389453, the annotated VP1 ORF starts at nt 261 (the first VP1-frame AUG codon), however, by homology with DQ388474, VP1 initiation likely (also) occurs at a GUG (context gauGUGu) codon aligning with the VP1 AUG codon in DQ388474. AUG2 itself overlaps the GUG codon in the +1 frame relative to the VP1 ORF and heads a 6-codon ORF terminating at a UAA codon which is separated from the ORFX AUG codons (adjacent AUG [34]) by a 12-nt spacer. As in DQ388474, AUG1 heads a 15-codon ORF that overlaps the VP1 ORF GUG codon and terminates just 5' of AUG3.

In AF291684 (CPV-15), ORFX has 62 codons (221..406; 7.8 kDa), and overlaps the VP1 ORF (34..4119) in the +1 reading frame. The VP1 ORF starts at AUG1 (context aguAUGu) but ORFX starts at AUG5 (context auaAUGc), with AUG [234] in the ORFX frame but heading two short ORFs: AUGaacUGAucaAUGaaaAUGaguuacUAG (nt coords 83..112).

In DQ087278 (APRV), ORFX has 87 codons (113..373; 10.2 kDa), and overlaps the VP1 ORF (34..3639) in the +1 reading frame. The VP1 ORF starts at AUG1 (context uuuAUGa) and ORFX starts at AUG3 (context aaaAUGa), with AUG2 (context agaAUGu) being in the VP1 frame, five codons 3' of AUG1. 

MLOGD can not be used effectively on an alignment of all eight sequences because the pairwise divergences are too great, so we can not robustly assess the coding potential of ORFX outside of CPV-1 with the currently available sequence data. However, the fact that the +1 frame ORF is present at the same alignment location in all eight sequences, even though the mean divergence of the 8-sequence alignment within the ORFX region is ~1.5 independent base variations per alignment nucleotide column, suggests that it is functionally important.

The genomic location of ORFX is more-or-less consistent with a leaky scanning model for ORFX translation, albeit perhaps at relatively low efficiency since the contexts of the VP1 initiation codons are not particularly weak. The frequent presence of an additional AUG codon, preceding both the ORFX and VP1 AUG codons, is a little confusing both for VP1 and ORFX translation though, in some cases, this AUG codon may play a role in moving some ribosomes past the VP1 initiation codon, allowing them to reinitiate at the ORFX AUG codon. There may also be other *cis*-elements that promote ORFX translation (although we were unable to locate candidate RNA secondary structures for this purpose). The presence of two short intervening ORFs argues against simple leaky scanning in CPV-15. It is interesting, and possibly relevant, that in another *Reoviridae *species – *Avian reovirus *– a novel, as yet not fully understood, scanning-independent ribosome migration mechanism is used to bypass two upstream CDSs in order to translate the 3'-proximal CDS on the tricistronic S1 mRNA [10].

In AF389462 (CPV-1), the ORFX peptide sequence is MKRNINNQKLTAVQIMEKERQEHAIKQLEILRLKRELEMKRKQVQALEDRLMARAVVEQMQK. With the exception of APRV, the 62–64-aa ORFX peptide sequence is very hydrophilic (≥ 60% of residues are polar) and basic (22–27% of residues are basic) with 13–16 Arg/Lys residues. The APRV ORFX peptide sequence is longer (87 aa) but has a similar hydrophobicity profile, is also basic, and contains 12 Arg/Lys residues. One potential function for ORFX product may be suppression of silencing via dsRNA binding (cf. [11]). Alternatively, the Arg/Lys residues may mediate nuclear localization. Application of blastp [8] to the eight ORFX peptide sequences revealed no similar amino acid sequences in GenBank (10 Apr 2008). Similarly, application of InterProScan [12] returned no hits (protein motifs, domains etc).

The VP1 protein itself (~150 kDa) has been identified as the major capsid protein [13,14]. It shares some homology with the *Oryzavirus *major capsid protein P3 [13] and, expressed independently, assembles into single-shelled virus-like particles [14]. Our analysis indicates that the 5'-terminal region of the VP1 ORF encodes, in the +1 reading-frame, an additional 7.5–7.8 kDa protein. Cypoviruses have potential uses in insect pest control and are also important pathogens of commercially important insects such as silkworms. Although much remains to be discovered about even the ten known cypovirus proteins, it is important to know of any additional proteins as early as possible. In particular, if an overlapping gene remains undetected, then its functions may be wrongly attributed to the gene that it overlaps, leading to persistent and wasteful confusion. We hope that presentation of this bioinformatic analysis will stimulate an attempt to experimentally verify the expression and functional role of ORFX product. Initial verification could be by means of immunoblotting with ORFX-specific antibodies, bearing in mind, however, that it may be expressed at relatively low levels.

## Competing interests

The authors declare that they have no competing interests.

## Authors' contributions

AEF carried out the bioinformatic analysis and wrote the manuscript. Both authors edited and approved the final manuscript.
